# On the Condition of the Mouth and Teeth during Pregnancy

**Published:** 1874-12

**Authors:** Oakley Coles


TO BE CONTINUED.

---

### ARTICLE V.

### *On the Condition of the Mouth and Teeth During Pregnancy.*

BY MR. OAKLEY COLES.

*Mr. President and Gentlemen:*—There is an old-fashioned phrase, common amongst a large section of the female community,—"for every child a tooth."

I believe that the accuracy of the statement contained in this old woman's fable may be fairly doubted, but it is nevertheless true, that the period of pregnancy is one in which the oral secretions are subject to considerable alterations, whilst the teeth are peculiarly liable at this time to decay, or to undergo pathological changes.

In the artificial life of the present day, the teeth are less able to resist the strain made upon them, in common with the other parts of the organism; hence we have during pregnancy an increasing liability to caries with each generation.

The interest of the subject, rather than my own qualification for writing on it, must be my excuse for treating of a matter in connection with which I would sooner have listened to some older members of this Society.

I propose in the following paper noticing—

1. The changes and general condition of the teeth during pregnancy.

2. The condition of the gums.

3. The oral secretions, with their changes and influence upon the teeth.

4. The neuralgia of pregnancy.

And lastly, the remedial agents useful during pregnancy.

1. *The changes and general condition of the teeth during pregnancy.*—In a large number of cases the period of pregnancy passes by in a perfectly normal condition, the processes that take place are physiological, and no evidence is given (in the mouth, at any rate) of any pathological process having occurred. In other instances we find the first few months of pregnancy accompanied with a severe toothache. This is usually (if it be a first pregnancy) attributable to neuralgia, and not to odontalgia. I shall therefore speak of it further on.

When the pain arises from the teeth, we generally find them the subjects of caries, though not always. The one variety that I have found most prevalent is the brown caries. The teeth are discolored over most of their surface, and the margin of the cavity of decay is black, and beyond the margin, a shading of brown from almost black to a stone color, spreads over the surface of the tooth adjoining the carious part. In the upper incisors and canines there is generally a line of green discoloration on the labial surface of the tooth, following the crescentic outline of the gum, or, in place of the green coloring matter, a dark line of blackish-brown, looking as if the enamel had been charred with a hot iron. The enamel is opaque in appearance and brittle to the touch.

As a rule, the upper and lower bicuspids will be seen in various stages of decay, the lateral incisors of the upper jaw standing next in liabity to caries, and then the central incisors; the lower incisors, as a rule, escape, as under ordinary circumstances, apart from pregnancy.

The women of the lower classes seem more liable to this special description of caries during and subsequently to pregnancy than those of the middle and upper sections of the community.

A second variety that is very interesting, but comparatively rarely seen, is where decay takes place on the prominences of the palatine cusps of the upper bicuspids and molars. The cavity presents a ragged margin, and does not generally give rise to pain, since its existence is soon noticed by the tongue of the patient, and relief sought for at the hands of the dental surgeon. The dentine is found softened, but not much discolored, nor is the enamel changed in appearance, beyond the opacity surrounding the outline of the cavity.

We shall see the special interest attaching to this peculiarity of situation when speaking of the etiology of caries during pregnancy.

There is still a third description of decay requiring a few words of reference, and that is the soft caries.

Near the margins of the gum and around the necks of the teeth the enamel seems either to have disappeared or to have become so softened in texture, that a sharp excavator or enamel chisel easily passes through it: the teeth are very often exquisitively sensitive and conscious of every thermal change. The removal of the softened tissue is an operation inflicting considerable pain, and it is very easy to go on cutting away till the pulp-cavity is nearly approached. Sometimes this softened state of the enamel and dentine is confined to a limited area, whilst in other instances it passes round the neck of the tooth like a ring. Whenever it is present, the enamel is generally less opaque in appearance than in the first variety of caries referred to, and there is, as a rule, no green or brown discoloration or deposit on the labial surface of the upper incisors.

All the teeth are liable to an attack of this process of disintegration; but the upper bicuspids, lower bicuspids and molars, and upper laterals and canines, seem somewhat more subject to it than the rest of the teeth. Such an observation, however, is but of small value unless extending over a large area of cases than one observer has the opportunity of seeing.

There is a modification of this last variety, in which we find a general softening of the tooth without any actual decay, as it is ordinarily understood. The whole tooth (most frequently in my own experience an upper bicuspid) becomes very sensitive to the touch. A draught of cold air gives severe pain, hot fluids taken into the mouth produce discomfort, and occasionally even an instrument covered with cotton wool, or the touch of a linen napkin, will give a severe shock to the patient's nerves. Later on, in the history of such a case, we find the tooth more loosened from its socekt, and at last it becomes a source of so much irritation to the patient, that even within a few weeks of her accouchment, she prefers undergoing the pain of extraction to the constant plague of an aching tooth.

Although we have loosening, there ts no great elongation or, to speak more correctly, protrusion from the socket, and we find that the loosening has been partly due to the absorption of the alveolus that has taken place. On removing the tooth, the periosteum of the fang is seen to be scanty in substance and anæmic in appearance. The tooth can be cut through easily with the excising forceps or hand-saw; and on examining the pulp, even with an ordinary magnifying-glass, and pressing it between the finger and thumb, we are able to observe its fatty condition, unmistakably distinguishable from the ordinary healthy or inflamed pulp.

The cut portion of the tooth has also a greasy surface, very different to that which is felt with any tooth after extraction for inflamed pulp or periosteum with alveolar abscess. We have, I believe, a condition that may be fairly and scientifically called "fatty degeneration"

Although, so far as I am aware, this pathological process has not hitherto been associated with the pregnant state, still it is one that has been fully recognized and described in connection with senile and other forms of decay, by Professor Wedl ("Dental Pathology," pp. 233, 241, 415.)

Thus, in treating the affections of the pulp he writes:—"Fatty degeneration is of frequent occurrence, and is manifested in a general way by its diminished volume and succulency, its recession and pale reddish discoloration, with a trace of yellow. * * * In some parts, the fat-globules form chainlike or fusiform aggregations, and follow the course of the vessels and nerves; in others the minute fat-molecules are scattered in the interstitial connective tissue, which may be cleared up by the addition of acetic acid or carbonate of soda. * * * The pulps of milk teeth also, while the latter are undergoing resorption during the period of dentition, and really are senescent teeth, sometimes become the subjects of the above fatty degenerations, which occur in the same manner and form as with the subsequent teeth." Writing again ot atrophy of the pulp, he states:—"The highest grade is displayed in the degeneration of the pulp into a soft greasy mass, unaccompanied by the odor of composition. * * * A tooth which has been the subject of a total decay of this description is no longer firmly attached within its alveolus." He says:—"It is a familiar fact, that dead animal tissues undergo a marked fatty degeneration under certain circumstances; * * * fat may also be deposited in dead dentine; indeed it is found interposed in the dentinal canals in the form of drops."

There is, however, another explanation that may be offered for the softening that takes place in otherwise sound teeth, during pregnancy, and that is either a diminution in the supply of earthy salts, to repair the waste of constant use, or an actual resorption of the calcareous constituents of the teeth, such as takes place during the progress of osteomalacia. Osteo-malacia is a disease peculiar to women, and generally connected with the pregnant or puerperal state, and it is quite possible that the condition of the teeth that we have under consideration may be due to a modification of such a pathological process. This hypothesis is further strengthened by the fact, that in some cases softening takes place without any appearanee of fatty degeneration.

It is somewhat singular, though I think it is capable of explanation, that "soft or white caries," with softening and loosening of the teeth unaffected with caries, is more commonly found amongst the upper classes, and those who have the reputation of being well nourished, than amongst the poor and lower middle class. It is perhaps desirable to mention, before leaving this part of the subject, that teeth that have been plugged before pregnancy are as frequently the seat of caries as others; and, moreover, we not unfrequently find that the margins of a cavity will have given way, leaving the plug standing alone, and only held in position by its deeply placed retaining points.

2. *The Conditions of the Gums.*—It may be well to state at once that, so far as my observations have gone, I have failed to discover any condition of the gums that can be regarded as specially and alone connected with the pregnant state. Whilst, therefore, speaking of those changes that take place in the gums, we must bear in mind that their appearance is due to the influences that are in operation at other times than during pregnancy, and that beyond the fact of their coincidence, they would call for no special attention.

"When the teeth are the subject of brown caries, "with green deposit on their crowns, and tartar around the necks and fangs, we generally find the margin of the gum raised, glistening and distended; immediately below the margin there is a well defined reddish-purple line; occasionally this is of so deep a purple tint that it has been mistaken for a symptom of lead poisoning; at other times we find midway between the margin of the gum and the purple line an intermediate space covered with cast-off epithelial cells. The surface of the gum is hot and painful to the touch, and bleeds readily. The affection is not confined to any special part of the dental margins, but is nevertheless most severe in the incisive region of both the upper and lower jaw.

In other cases we find the gums of a rather full color, with their margins rounded, but not very much thickened; they are, however, separated from their attachment to the necks of the lower teeth (the incisors, canines, and bicuspids especially,) and on pressing gently from below upwards, on the anterior surface of the gum, a thin fluid, and very often pus, is seen to ooze from around the neck of the tooth and free border of the gum.

This condition is not confined to those who are negligent in the care of their teeth, but will also be found in mouths where the tooth brush is used both freely and frequently.

We have here, doubtless, a state of passive congestion owing to impeded circulation, in the course of which a serous or corpuscular transudation takes place through the walls of the distended veins.

Associated (most frequently) with white caries, or softening and loosening of the teeth, we have an anæmic condition of the gums. Here, instead of the margins being thick and rounded, they are thin, pale and shriveled in appearance, withdrawn from the necks of the teeth, yet still adherent at a higher point of attachment; not unfrequently there is seen a prominent ridge a little beyond the free border, and this is sometimes of a deeper tint than the surrounding membrane. If any condition of the gums could be said to be especially associated with pregnancy, it would be that which I have just described, more especially as it is notably present in cases of frequently-recurring pregnancy.

3. *The Oral Secretions, with their Changes and Influence upon the Teeth.*—The changes that take place in the oral and buccal secretions are probably the most potent agents in the production of caries during pregnancy. The secre tion of saliva is increased to a great extent, in many cases by irritation of the second and third branches of the fifth pair of nerves, and also by a stimulation derived from the acrid eructions of the stomach and the morning sickness during the early months of pregnancy.

Test papers, applied early in the morning, will determine the acidity of the contents of the mouth in a large majority of the cases examined, especially in first pregnancies; later in the day it is difficult to obtain data by means of test papers that are of any value, since there are many agencies at work tending to counteract (if present) the acidity of the saliva. Patients will tell you that during their first two pregnancies (in most cases) they awake with an acid taste in the mouth, and after the sickness that usually occurs, the acidity in the mouth is much more pronounced, whilst the vomited matter is so acid in its character, that, even after standing for a considerable time, or if treated at once, by the addition of a little carbonate of soda or potash, a free liberation of carbonic acid takes place.

Before the sickness the secretion of the saliva and mucus is generally increased, and the flow continues under the influence of the acid principle arising from the stomach.

From the recent investigations that have been made, there can be little doubt, I think, that the most fruitful source of the excessive flow of saliva is the acid matter regurgitated from the stomach, which undoubtedly possesses the power of stimulating the salivary glands. This is a point of great interest in relation to the origin of caries; I must, therefore, enter into the matter more fully than might at a first thought seem needful.

Dr. Sidney Ringer, in his "Handbook of Therapeutics" (p. 78,) says:—"It has been fully established by repeated and careful experiments, that dilute acids, when taken into the stomach, possess the power to check its secretion; while on the other hand, alkalies stand prominent among the most powerful exciters of the secretion of the gastric juice. From these facts the more general law has been inferred—

namely, that acids possess the power to check the production of acid secretions from glands, while they increase the flow of alkaline ones, while the very reverse is the case with alkalies, which are supposed to check alkaline but to increase acid secretions. This general law (says the author) is made the more probable, as it fully explains the effects, which experience has shown to be true, of acids on the secretions of the alimentary canal in disease."

We see from the foregoing extract that there is more than one way in which to account both for acidity of the stomach and increased flow of saliva. The salivary secretion may arise from dental irritation or neuralgia; and I may here mention that this has its analogue in the increased secretion that flows from lachrymal glands when the opthalmic division of the fifth is the seat of irritation. This alkaline saliva being swallowed would increase the flow of the gastric fluids, whilst acrid eructations following, we have the salivary glands still further stimulated.—*Odontological Society of Great Britain Transactions.*

